# Sex‐dependent cholinergic effects on amyloid pathology: A translational study

**DOI:** 10.1002/alz.13481

**Published:** 2023-10-17

**Authors:** Liliana German‐Castelan, Hayley R. C. Shanks, Robert Gros, Takashi Saito, Takaomi C. Saido, Lisa M. Saksida, Timothy J. Bussey, Marco A. M. Prado, Taylor W. Schmitz, Vania F. Prado, the Australian Imaging Biomarkers and Lifestyle flagship study of ageing

**Affiliations:** ^1^ Robarts Research Institute Schulich School of Medicine and Dentistry University of Western Ontario London Ontario Canada; ^2^ Neuroscience program Schulich School of Medicine and Dentistry University of Western Ontario London Ontario Canada; ^3^ Department of Medicine Schulich School of Medicine & Dentistry University of Western Ontario London Ontario Canada; ^4^ Department of Physiology and Pharmacology Schulich School of Medicine & Dentistry University of Western Ontario London Ontario Canada; ^5^ Department of Neurocognitive Science Institute of Brain Science Nagoya City University Graduate School of Medical Sciences Nagoya Japan; ^6^ Laboratory for Proteolytic Neuroscience RIKEN Center for Brain Science Wako, Saitama Japan; ^7^ Western Institute for Neuroscience University of Western Ontario London Ontario Canada; ^8^ Department of Anatomy and Cell Biology Schulich School of Medicine & Dentistry University of Western Ontario London Ontario Canada; ^9^ Lawson Health Research Institute St. Joseph's Hospital London Ontario Canada

**Keywords:** amyloid pathology, cholinergic system, humanized App mouse models, MRI, ovariectomy, PET, sexual dimorphism, translational study

## Abstract

**INTRODUCTION:**

About two‐thirds of Alzheimer's Disease (AD) patients are women, who exhibit more severe pathology and cognitive decline than men. Whether biological sex causally modulates the relationship between cholinergic signaling and amyloid pathology remains unknown.

**METHODS:**

We quantified amyloid beta (Aβ) in male and female App‐mutant mice with either decreased or increased cholinergic tone and examined the impact of ovariectomy and estradiol replacement in this relationship. We also investigated longitudinal changes in basal forebrain (cholinergic function) and Aβ in elderly individuals.

**RESULTS:**

We show a causal relationship between cholinergic tone and amyloid pathology in males and ovariectomized female mice, which is decoupled in ovary‐intact and ovariectomized females receiving estradiol. In elderly humans, cholinergic loss exacerbates Aβ.

**DISCUSSION:**

Our findings emphasize the importance of reflecting human menopause in mouse models. They also support a role for therapies targeting estradiol and cholinergic signaling to reduce Aβ.

**Highlights:**

- Cholinergic tone regulates amyloid beta (Aβ) pathology in males and ovariectomized female mice.
- Estradiol uncouples the relationship between cholinergic tone and Aβ.
- In elderly humans, cholinergic loss correlates with increased Aβ in both sexes.

## 1 BACKGROUND

Alzheimer's Disease (AD) is the leading cause of dementia, affecting about 50 million people worldwide.^1^ Approximately two‐thirds of late‐onset AD (LOAD) patients are women, who also exhibit more severe pathology and accelerated cognitive decline compared to men.^2^, ^3^ Age‐related hormonal changes have been proposed to explain the sexual dimorphism in LOAD risk.^3^ Female menopause causes a significant decline in estrogen signaling during the 4th and 5th decades of life and because estrogens are neurotrophic and neuroprotective, their sudden loss is thought to increase susceptibility to age‐related pathophysiology and neurodegeneration.^4^, ^5^ Together, these findings imply that the sexual dimorphism in LOAD is likely caused by sex‐dependent hormonal influences on neuronal vulnerability and pathophysiology.

Several studies in mouse models of AD and in cognitively normal older adults at risk for AD have consistently pointed to the selective vulnerability of cholinergic neurons to amyloid pathology as an early and critical component of presymptomatic disease which predicts subsequent neurodegenerative progression.^6^, ^7^, ^8^ Indeed, basal forebrain cholinergic neurons dysfunction and degeneration are early pathological events in AD^9^, ^10^ that precede and predict cortical degeneration, clinical onset, and dementia severity.^10^, ^11^ There is also a close relationship between early dysfunctions in cholinergic signaling and amyloid β (Aβ) pathology. Decreased cholinergic signaling is associated with increased Aβ levels in the brain of mouse models and human patients.^6^, ^12^, ^13^, ^14^ Furthermore, Aβ reduces acetylcholine (ACh) synthesis and release both in vitro and in vivo.^15^, ^16^ Given that the brain cholinergic system of males and females show subtle functional differences and that sex hormones exert trophic effects on the cholinergic system,^17^ we hypothesized that biological sex may causally influence the relationship between cholinergic tone and amyloid pathology.

In this study, we took a cross‐species translational strategy to test this hypothesis. We used existing mouse models in which we genetically targeted the vesicular acetylcholine transporter (VAChT), a protein that is crucial for ACh release and signalling.^7^ These models included mice with decreased forebrain VAChT expression, thus modeling basal forebrain cholinergic dysfunction in AD,^6^, ^18^, ^19^ and mice with increased VAChT expression.^20^ We crossed these VAChT models with amyloid precursor protein (*A* *p* *p*) knock‐in (KI) mice that recapitulate several characteristics of the AD pathophysiology seen in humans and do not overexpress APP, avoiding the expression of artificial peptides and phenotypes.^21^ Critically, each experiment was run separately for male and female mice, and we also examined the impact of surgically induced menopause and pharmacological replacement of estradiol. Additionally, we utilized longitudinal multimodal neuroimaging to examine the interactions of cholinergic tone and amyloid pathology in cognitively normal older adults. To do so, we tracked changes in basal forebrain gray matter volume, quantified from structural magnetic resonance imaging (MRI), time‐locked over a 2‐year interval to corresponding changes in cerebral amyloid accumulation quantified from positron emission tomography (PET). As in the mouse experiments, we explicitly examined the effect of sex on these interactions.

**RESEARCH IN CONTEXT**

1. **Systematic review**: Alzheimer's disease (AD) poses a significant burden on women as they account for two‐thirds of all cases, show faster cognitive deterioration, and more severe pathology. Loss of sex hormones during menopause may contribute for the sexual dimorphism. Whether biological sex influences the relationship between cholinergic signaling and amyloid beta (Aβ) pathology is unknown.
2. **Interpretation**: Our findings in mice indicate that in young individuals, there is an inverse relationship between cholinergic signaling and Aβ accumulation in males, but not in females, as estradiol uncouples the relationship. In elderly humans, after estradiol production has been absent in women for several years, the cholinergic‐signaling/Aβ‐accumulation inverse relationship is observed but is not influenced by sex.
3. **Future directions**: We propose that estradiol suppression is necessary in preclinical studies to better reflect the hormonal context after menopause. Additionally, we emphasize the need for large multimodal longitudinal imaging human studies covering pre‐ and peri‐menopausal ages.

Our results show an inverse relationship between VAChT levels and Aβ pathology in males and ovariectomized female mice. Human data analyses revealed that cholinergic dysfunction exacerbates amyloid pathology in both men and postmenopausal women. Interestingly, ovary‐intact or estradiol‐treated ovariectomized females displayed a decoupled relationship between cholinergic tone and amyloid, suggesting an estradiol‐mediated effect. These findings demonstrate a moderating effect of estradiol on early AD pathophysiology and highlight the importance of modelling postmenopausal hormonal state in female mice to improve the translational validity and more accurately reflect the outcomes observed in women with AD.

## 2 METHODS

### 2.1 Study design

We investigated whether biological sex affects the relationship between VAChT and plaque deposition in AD mouse models and elderly humans. We used humanized App‐KI mice carrying AD‐associated familial mutations^21^ that were crossed with mice either lacking^6^, ^18^, ^19^ or overexpressing VAChT^20^ in the brain. VAChT levels were determined in the cortex by immunoblots. Amyloid plaques and insoluble Aβ were analyzed by immunofluorescence and enzyme‐linked immunosorbent assay (ELISA). Male and female mice were investigated. The number of mice used were determined based on previous published studies. For all experiments at least three technical replicates were performed. For mice, allocation to experimental group (controls vs mutant) was driven by Mendelian inheritance. For treatments and surgical procedures, animals were randomized by block randomization. The researcher was blind to sex/treatment/genotype during tissue collection and histological and amyloid quantification analyses. Outliers were identified by the Grubbs’ test and excluded from the dataset before analysis. MRI and amyloid PET data from cognitively normal, healthy volunteers (58 males, 72 females; ∼75 years old) were analyzed.

### 2.2 Animals

Use and care of animals was conducted in agreement with the Canadian Council of Animal Care guidelines and the Animal Use Protocols approved by the University of Western Ontario (2020‐162 and 2020‐163). VAChT^flox/flox^ and forebrainVAChT‐knockout (for simplicity named VAChT‐KO; VAChT^Nkx2.1‐Cre‐flox/flox^) mouse lines generation has been described.^18^, ^22^ VAChT‐overexpressing mice (ChAT‐ChR2‐EYFP) were obtained from Jackson Laboratory (B6.Cg‐Tg[Chat‐Cop4*H134R/EYFP]6Gfng/J).^20^ *App^NL/NL^*, *App^NL‐F/NL‐F^*, and *App^NL‐G‐F/NL‐G‐F^* mice, with Swedish, Arctic, and Beyreuther/Iberian mutations, were described previously.^21^

*App^NL‐F/NL‐F^* and VAChT‐KO mouse lines were crossed to generate *App^NL‐F/NL‐F^*‐VAChT‐KO mice, resulting in *App^NL‐F/NL‐F^* mice with decreased cholinergic tone.

*App^NL‐G‐F/NL‐G‐F^* and ChAT‐ChR2‐EYFP mouse lines were crossed to generate *App^NL‐G‐F/NL‐G‐F^* mice overexpressing VAChT (*App^NL‐G‐F/NL‐G‐F^*‐ VAChT^over^), showing a hypercholinergic state. Mice were exposed to 12‐hour light/12‐hour dark cycles and received ad‐libitum access to regular chow and water. Mice were housed in plexiglass cages with 1 to 3 littermates. Room temperature (RT) and humidity were controlled at 22 to 25°C and 40% to 60% respectively.

### 2.3 Mouse brain fractionation for ELISA and Western blot

Mice were anesthetized with ketamine (100 mg/kg)‐xylazine (25 mg/kg) in sterile saline (0.9% sodium chloride) and euthanized by transcardial perfusion with ice‐cold phosphate‐buffered saline (PBS). Brains were harvested and one hemisphere was dissected into different regions and snap‐frozen on dry ice before transferring to −80°C for storage while the other hemisphere was collected for immunofluorescence.

To prepare the protein extracts, cortices were homogenized in 300 μL of Tris buffer (50 mM Tris‐HCl, 200 mM NaCl, 2 mM Na‐EDTA, pH 7.2) with phosphatase inhibitors (1 mM NaF and 0.1 mM Na_3_VO_4_) and protease inhibitor cocktail (Calbiochem, catalog# 539134‐1SET, 1:100). Homogenates were centrifuged for 1 h at 100,000 × g at 4°C. Supernatants (Tris‐soluble fraction) were stored at −80°C. Pellets were resuspended in 300 μL of RIPA buffer (50 mM Tris, 150 mM NaCl, 0.1% SDS, 0.5% sodium deoxycholate, 1% triton X‐100, pH 8.0) with phosphatase and protease inhibitors. Samples were centrifuged at 16,000 × g for 40 min at 4°C. Supernatants (RIPA‐soluble fraction) were stored at −80°C for western blotting. Pellets were resuspended in 600 μL of 5 M guanidine‐HCl, 50 mM HEPES, 5 mM EDTA, pH 7.3, followed by rotation at 4°C overnight (insoluble fraction) and were stored at −80°C for use in ELISA.

### 2.4 Western blotting

Immunoblotting was performed with the RIPA‐soluble fraction (described above). Protein quantification was done using the DC protein assay (BioRad, catalog# 5000112). Twenty‐five micrograms of protein were separated on 4% to 12% Bis‐Tris Plus Gels (Thermo Fisher) and transferred onto PVDF membranes (EMD Millipore, Catalog# IVPH00010) with a BioRad Trans‐Blot Turbo system. Antibodies were anti‐VAChT (Synaptic System, catalog# 139103, 1:2000), anti‐synaptophysin (Millipore‐Sigma, catalog# S5768, 1:3000), anti‐synaptophysin (Abcam, catalog# ab8049, 1:2000), anti‐mouse HRP (Sigma, catalog# SAB3701095, 1:5000), and anti‐rabbit HRP (BioRad, catalog# 170‐6515, 1:7500). Protein bands were visualized using chemiluminescence in the ChemiDoc MP Imaging system (BioRad). Quantification of the bands was obtained by densitometric analysis using the ImageLab software (BioRad).

### 2.5 Immunofluorescence

Mice were transcardially perfused with PBS and one hemisphere was post‐fixed with 4% paraformaldehyde for 24 h at 4°C. Tissue was washed and stored in PBS with 0.02% sodium azide at 4°C until sectioning using a vibratome. Free‐floating sagittal sections (30 μm) were washed in PBS for 10 min and then heated to 80°C in 10 mM sodium citrate, pH 6.1, for 30 min for antigen retrieval and then cooled at RT for 30 min. Sections were then washed three times for 5 min in Tris‐buffered saline (TBS) and permeabilized in TBS + 3% Triton‐X twice for 5 min. Sections were blocked using 5% goat serum, 0.5% triton‐X, and 2% bovine serum albumin (BSA) in TBS for 90 min at RT and then incubated with primary antibodies: anti‐β‐amyloid clone 6e10 (Biolegend, catalog# 803001, 1:200), anti‐VAChT (Synaptic System, catalog# 139103, 1:500), and anti‐mCherry (Abcam, catalog# ab 205402, 1:1000) at 4°C. After ∼18‐h incubation, sections were washed twice for 5 min with TBS and incubated with secondary anti‐mouse Alexa Fluor 546 (Thermo Fisher Scientific, catalog# A10036, 1:500), anti‐rabbit Alexa Fluor 488 (Thermo Fisher Scientific, catalog# A11034, 1:500), and anti‐chicken Alexa Fluor 633 (Thermo Fisher Scientific, catalog# A21103, 1:500) for 90 min at RT in the dark. Sections were washed twice with TBS and incubated with Hoechst 33342 (Thermo Fisher Scientific, catalog# 62249, 1:1000) for 10 min. After the 10‐min wash, sections were mounted on slides using Immu‐Mount (Thermo Fisher Scientific, catalog# 9990402). Images were captured using EVOS FL Auto 2 Cell Imaging System (Invitrogen) and Leica DM6B Thunder imager (Leica Microsystems Inc).

### 2.6 Ovariectomies

Mice were bilaterally ovariectomized or subjected to sham surgery when 4‐ to 5‐weeks‐old. Following anesthesia with isoflurane (2% to 2.5%), each mouse was placed in sternal recumbency and the skin was disinfected with ethanol and iodine. A midline incision (∼1 cm in length) was made in the mid‐dorsum and skin was bluntly dissected from the underlying muscle. Location of the ovary was made by visualizing the ovary‐surrounding fat pad, on the flanks of the animal, caudal to the kidneys. A small incision in the muscle directly above the ovaries was made, and the ovary was pulled out using forceps. A single ligature was placed around the oviduct, the ovary was removed, and the uterine horn was returned into the abdominal cavity. Muscle incisions were closed using absorbable suture and skin incision was closed with one staple. For pain management, 5 mg/kg Metacam was administered intraperitoneally while animals were anesthetized and then every 24 h up to 48 h postoperatively. Mice were allowed to recover in a clean cage with supplemental heat and then returned to the home cage. Post‐surgery, mice were provided with moistened chow and water on the cage bottom. Staples were removed 7 to 10 days after surgery. Sham mice underwent the same surgical procedure, except for the removal of ovaries. At the end of the experimental period, the uteri were dissected, dried overnight at 37°C, and weighed.

### 2.7 Hormone replacement

A subgroup of ovariectomized females received subcutaneous pellets of either estradiol (E2) (Belma Technologies, catalog# E2L‐M/60) or placebo (Belma Technologies, catalog# E2‐M‐Placebo). Immediately after ovariectomy (OVX), the skin of the original incision was bluntly dissected in a cephalic direction. A tunneller containing the pellets was inserted and the implants were deposited in the scapular region. The incision was closed with one staple and animals followed the postoperative procedure described above. The E2 implants release 0.7 to 1.3 mg E2/24 h for plasma concentrations of 35.2 to 72.0 ng/mL. Animals were perfused 4 weeks after surgery.

### 2.8 Quantification of amyloid burden

Tissue sections were quantified using ImageJ software (National Institutes of Health). Pictures were converted to 8‐bit gray scale and Aβ deposits were segmented by applying a binary threshold, to obtain an image with a white background and black amyloid plaques. Aβ burden was quantified as the percentage of the entire cortex occupied by amyloid deposits. Three to four sections per mouse were averaged and sections from 3 to 4 mice per group were used. Experimenter was blind to sex and genotype during image collection and analysis.

### 2.9 Human Aβ 42 ELISA

ELISA for the guanidine‐insoluble Aβ fraction obtained from cortical extracts was performed using the Amyloid beta 42 Human ELISA kit (Thermo Fisher, catalog#KHB3441) following the manufacturer's instructions.

### 2.10 Stereotaxic surgery and adeno‐associated virus (AAV) infusion

Nine‐month‐old *App^NL‐F^* mice were anesthetized with isoflurane (2% to 2.5%) and placed in a stereotaxic frame. A heating pad was placed under the mice to keep a body temperature of 37°C. The head was disinfected with chlorhexidine 0.5% and the top of the skull was exposed. A hole was made in the skull using a hand drill. An infusion of adeno‐associated virus (AAV) was made by injecting undiluted AAV9‐eSyn.mCherry‐2A‐mSLC18A3‐WPRE (1.3 × 10^13^ genome copies (GC)/mL, Vector Biosystems Inc) in the basal forebrain using a microsyringe pump (0.6 μL total volume at a rate of 0.2 μL per minute) at the coordinates AP: 0.85 mm, ML: 0.0 mm, DV 4.85 mm from Bregma.^23^ A separate cohort received undiluted AAV9‐hSyn‐mCherry (1.3 × 10^13^ GC/mL, Addgene viral prep #114472‐AAV9; RRID: Addgene_114472) as a control. The injection system was left in place for 6 min and then slowly withdrawn. Skin incision was sutured and 5 mg/kg Metacam was administered intraperitoneally while the animals were still anesthetized and then every 24 h up to 48 h postoperatively. Mice were allowed to recover in a clean cage with supplemental heat and then returned to the home cage. Post‐surgery mice were provided with moistened chow and water on the cage bottom.

### 2.11 Human data

#### 2.11.1 Participants

Human samples consisted of older adults from the Australian Imaging, Biomarker & Lifestyle (AIBL) Flagship Study of Aeging (www.aibl.csiro.au).^24^ For inclusion in our study, AIBL participants needed to be cognitively normal at baseline visit, have longitudinal 3 Tesla structural MRI, and have longitudinal amyloid PET. AIBL amyloid PET data spanned multiple radiotracers: Pittsburgh compound B (^11^C‐PiB), ^18^F‐Flutemetamol and Florbetapir (^18^F‐AV‐45). For participants with amyloid PET data using multiple radiotracers, the radiotracer with the largest number of scans was chosen. Our final sample consisted of 130 participants (Table 1; 58 males, 72 females).

**TABLE 1:** Participant characteristics.

	Male	Female	Statistic	p‐value
*N*	58	72		
Median age	75 (64–92)	75.5 (65–91)	Z = −0.04	0.97
%Converters	13.8%	9.7%	χ^2^ = 0.52	0.58
%*APOE*4 carriers	27.6%	23.6%	χ^2^ = 0.28	0.68
Median MRI interval (years)	3.2 (0.8–6.3)	2.05 (1.2–6.3)	Z = 0.52	0.59
Median PET interval (years)	4.5 (1.3–6.1)	2.98 (1.3–6.4)	Z = 1.49	0.14

*Note*. AIBL participants information. Data are presented as median (range) unless otherwise specified. Wilcoxon rank sum test (Z) and chi‐square tests were used to assess group differences. *p < 0.05; **p < 0.01; ***p < 0.0001.

Abbreviations: AIBL, Australian Imaging, Biomarker and Lifestyle Study of Aging; APOE, apolipoprotein‐E; MRI, magnetic resonance imaging; PET, positron emission tomography.

#### 2.11.2 MRI preprocessing

Analysis of imaging data was performed in MATLAB's SPM12 (https://www.fil.ion.ucl.ac.uk/spm/software/spm12/). Raw T1 weighted MRI scans were downloaded from AIBL and coregistered to a template image. For each subject, serial longitudinal registration of all MRI scans was performed as described^25^ to create a symmetric midpoint average image. At this stage, one Jacobian determinant per timepoint was written out for each subject, which shows how much each voxel has been stretched or compressed at that time relative to the midpoint average.^25^ Each subject's midpoint average was segmented into tissue compartments in the SPM toolbox CAT12 using tissue probability maps which have been enhanced to improve subcortical grey matter classification.^26^ Native space grey matter segmentations from each subject's midpoint average image were multiplied by each of their Jacobian determinants to produce longitudinally modulated grey matter images which can be used to estimate grey matter volume at each timepoint. Grey matter segmentations across all participants were used in combination with geodesic shooting to create an AIBL‐specific population template which is representative of older adult brains. After AIBL template creation, each subject's longitudinally modulated grey matter segments were transformed into the AIBL template space. We defined two regions of interest in the structural MRI data: the whole brain grey matter and the posterior basal forebrain (nucleus basalis of Meynert and nucleus subputaminalis of Ayala). The whole brain grey matter was defined using the grey matter segment of the AIBL population template, binarized at a probability of *p ≥*  0.5. The posterior basal forebrain region of interest was defined using a stereotaxic map created through postmortem histology^27^ using the same methods we have reported in our previous work.^28^, ^29^ After definition of the whole brain and posterior basal forebrain masks, the get_totals.m function (http://www0.cs.ucl.ac.uk/staff/g.ridgway/vbm/get_totals.m) was used along with the longitudinally modulated grey matter segments in template space to estimate grey matter volume of each region at each time.

#### 2.11.3 PET preprocessing

Raw PET scans were obtained from AIBL. For each subject, PET data were aligned across all timepoints using SPM's realign. PET data were then coregistered and resliced to the structural MRI midpoint average. A cerebellar mask created with the Desikan‐Killiany atlas^30^ was used to define cerebellar grey and white matter in the AIBL population template space. This mask was reverse warped into each subject's native space. Each PET scan was normalized to its mean radiotracer uptake in the cerebellar mask. Next, PET scans were warped without modulation into AIBL template space to facilitate comparisons across subjects. Finally, PET scans were smoothed with an 8‐mm isotropic gaussian kernel.

#### 2.11.4 Quantification of longitudinal change

Longitudinal changes in structural MRI and PET data were quantified for each participant based on two timepoints of data for each modality. For each subject, we chose the two MRI scans with the longest interval and the two PET scans with the longest interval. We then quantified changes within each imaging modality using the following annual percent change (APC) formula:

$$APC\;=\left[{\left(\frac{final}{baseline}\right)}^{\frac{365}{time\;difference\;\left(days\right)}}-1\right]\;\;x\;100$$

In the APC formula, the final and baseline values are either the normalized voxel intensity of a PET image, or the grey matter volume of a region of interest. The annual percent change formula was chosen as our measure of longitudinal change as it allows for the standardization of time interval across participants.

### 2.12 Statistical analyses

All mouse data were statistically analyzed using GraphPad Prism 9 software. Unpaired, two‐tailed Student *t* test was used to compare two experimental groups, while two‐way analysis of variance (ANOVA) with appropriate post‐hoc test was utilized for multivariate two group comparisons.

For whole brain PET analyses, a voxel‐wise full factorial model was implemented in SPM12. In the factorial model, sex was modelled as a factor, and whole brain grey matter PET APC maps as the dependent variable. Age, *APOE* genotype, and PET radiotracer were included as covariates in the model. Posterior basal forebrain APC was also included as a covariate with an interaction with the sex factor to allow for the assessment of interactions between sex and basal forebrain degeneration on amyloid PET. We corrected the resulting maps for multiple comparisons using the family‐wise error rate correction in SPM.

Structural MRI region of interest APC values were not normally distributed. We established whether there was significant longitudinal change in grey matter regions of interest using the Wilcoxon signed‐rank test. Then, we compared the APC of each region in males and females using a two sample Wilcoxon rank sum test.

## 3 RESULTS

### 3.1 Increased VAChT ameliorates amyloid pathology in *App^NL‐G‐F^* males but not in ovary‐intact *App^NL‐G‐F^* females

**FIGURE 1:**
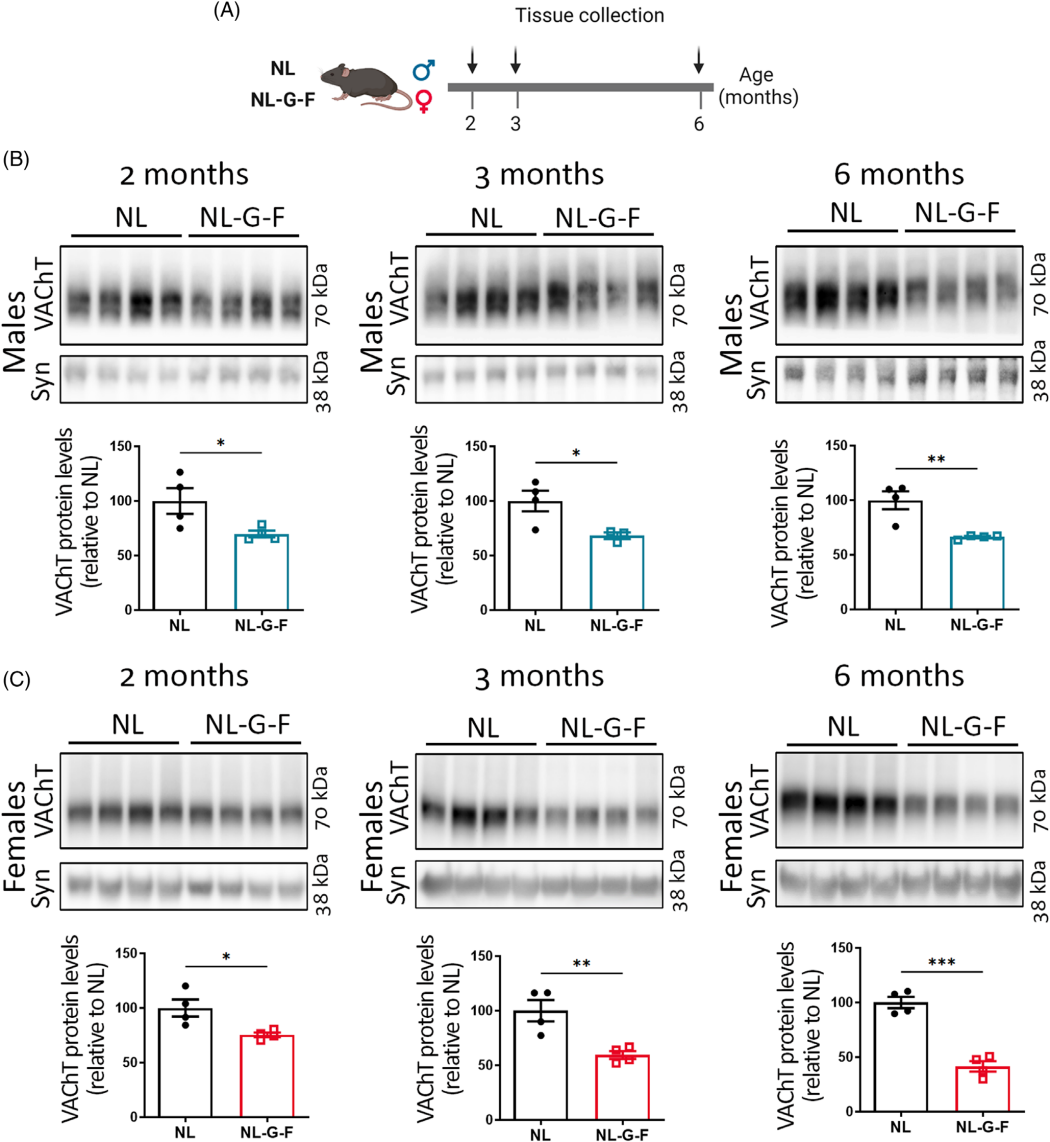
Vesicular acetylcholine transporter (VAChT) levels are decreased in the cortex of *App^NL‐G‐F^* mice. (A) Experimental timeline. (B‐C) VAChT quantification on *App^NL^* and *App^NL‐G‐F^* mice at 2, 3, and 6 months of age. Cortical samples from (B) males and (C) females. Synaptophysin (syn) was used as loading control. Each symbol in the graphs represents an individual animal. One outlier value was excluded from the 3‐month‐old male dataset. Data expressed as mean ± SEM. Unpaired, two‐tailed Student *t* test. *N* = 4. *p < 0.05, **p < 0.01, ***p < 0.001.

We utilized humanized amyloid precursor protein knock‐in mice (APP‐KI), including *App^NL^*, harboring the Swedish mutation; *App^NL‐F^*, containing the Swedish and the Iberian mutations; and *App^NL‐G‐F^*, carrying the Swedish, Iberian, and Arctic mutations.^21^ These models recapitulate AD pathophysiology seen in humans without overexpressing APP, avoiding artificial peptides and phenotypes.^21^ *App^NL‐F^* and *App^NL‐G‐F^* mice present age‐dependent amyloid plaque deposition, gliosis, and memory impairment, while *App^NL^* mice do not develop Aβ plaques and were used as controls. *App^NL‐F^* mice take around 9 months to develop plaque pathology while the more aggressive *App^NL‐G‐F^* model shows amyloid deposition as early as 2 months of age.^21^, ^31^ We initially tested whether VAChT protein levels are affected in the cortex of *App^NL‐G‐F^* compared to *App^NL^* mice (Figure 1A). Immunoblot analysis of cortical homogenates from 2‐, 3‐, and 6‐month‐old mice showed a significant decrease in VAChT levels in both male and female *App^NL‐G‐F^* mice compared to *App^NL^* at all three time points (Figure 1B, C). These data indicate that cholinergic deficiency arises early in this aggressive model of pathology.

**FIGURE 2:**
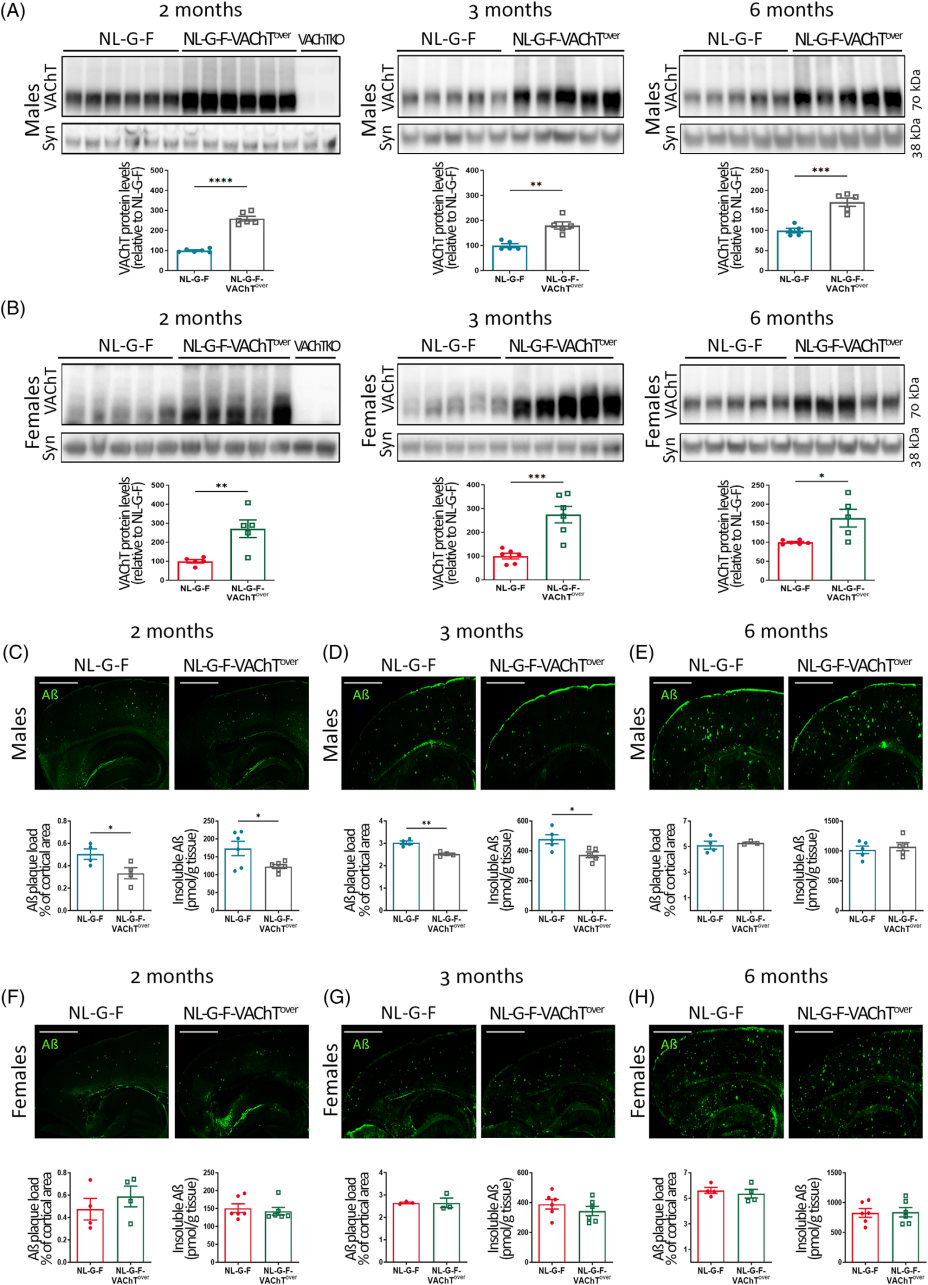
Vesicular acetylcholine transporter (VAChT) overexpression mitigates amyloid pathology in *App^NL‐G‐F^* males but not in ovary‐intact females. (A,B) VAChT levels in the cortex of 2‐, 3‐, and 6‐month‐old *App^NL‐G‐F^*‐VAChT^over^ (A) males and (B) females compared to *App^NL‐G‐F^* controls. *N* = 5 to 6. (C‐H) Plaque burden and insoluble amyloid beta (Aβ) in the cortex of *App^NL‐G‐F^* and *App^NL‐G‐F^*‐VAChT^over^ animals. Males (C) 2 months old, (D) 3 months old, and (E) 6 months old. *N* = 4 to 6. Females (F) 2 months old, (G) 3 months old, and (H) 6 months old. *N* = 3 to 6. Scale bars = 1000 μm. VAChT‐KO was added as negative control. Synaptophysin (syn) was used as loading control. Each symbol in the graphs represents an individual animal. Data are expressed as mean ± SEM. Unpaired, two‐tailed Student *t* test. *p < 0.05, **p < 0.01, ***p < 0.001, ****p < 0.0001.

To determine whether VAChT levels are causally associated with amyloid pathology, we crossed *App^NL‐G‐F^* animals with mice overexpressing VAChT (VAChT^over^)^20^, ^32^ to generate homozygous *App^NL‐G‐F^* mice overexpressing VAChT (*App^NL‐G‐F^*‐VAChT^over^). Immunoblot analysis of cortical extracts showed that VAChT levels are ∼two‐ to threefold higher in *App^NL‐G‐F^*‐VAChT^over^ mice when compared to *App^NL‐G‐F^* controls in both males and females at 2, 3, and 6 months of age (Figure 2A, B). Antibody specificity was confirmed by utilizing brain tissue from forebrain VAChT‐KO mice.^18^ In 2‐ and 3‐month‐old *App^NL‐G‐F^* males, higher VAChT levels effectively decreased amyloid deposition, evidenced by decreased plaque staining in cortical slices and reduced insoluble Aβ measured by ELISA (Figure 2C, D), suggesting that increasing VAChT levels can decrease pathology in this aggressive AD mouse model. However, upon aging (6 months old), the protective effect of increased VAChT expression over plaque deposition was lost, as both *App^NL‐G‐F^*‐VAChT^over^ mice and *App^NL‐G‐F^* controls showed similar amounts of Aβ immunofluorescence and insoluble Aβ levels (Figure 2E). Converging factors may be contributing for this “loss‐of‐effect.” That is, as *App^NL‐G‐F^* mice show age‐dependent VAChT deficiency, older (6‐month‐old) *App^NL‐G‐F^* mice have less VAChT‐overexpression than 2‐ to 3‐month‐old mice (Figure 2A‐B) and therefore have less cholinergic protection. Furthermore, the Aβ peptide present in *App^NL‐G‐F^* mice contains the Arctic mutation (E693G). This Aβ is resistant to proteolytic degradation^33^ and more susceptible to aggregation.^34^ Thus, as *App^NL‐G‐F^* mice age, the elevated Aβ levels have the potential to overwhelm the cholinergic protective mechanisms. In contrast, in females, neither plaque staining nor insoluble Aβ levels differed between *App^NL‐G‐F^*‐VAChT^over^ mice and controls at any of the time points (Figure 2F‐H). Notably, the amount of insoluble Aβ in males and females of the same age and genotype is similar (Table S1), suggesting that major differences in Aβ levels do not contribute to the protective effect of VAChT overexpression observed in males. These results suggest that there is a sexual dimorphism in the cholinergic regulation of plaque pathology.

### 3.2 Increased VAChT ameliorates amyloid pathology in ovariectomized *App^NL‐G‐F^* females, and the effect is blocked by estradiol

**FIGURE 3:**
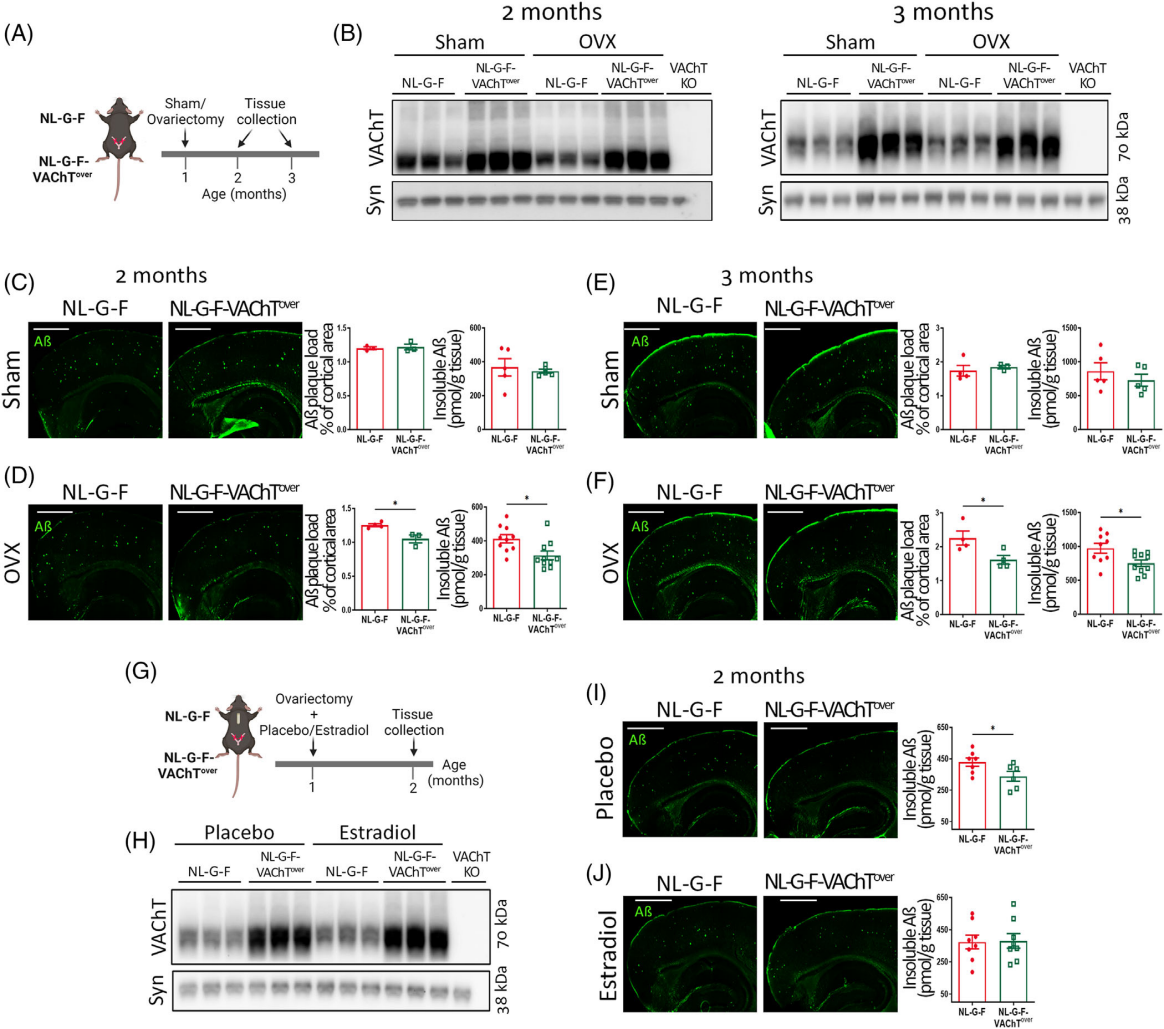
Ovariectomy (OVX) decreases amyloid pathology in *App^NL‐G‐F^*‐VAChT^over^ females, an effect blocked by estradiol. (A) Experimental timeline. (B) Vesicular acetylcholine transporter (VAChT) expression in the cortex of 2‐ and 3‐month‐old OVX‐*App^NL‐G‐F^* and OVX‐*App^NL‐G‐F^*‐VAChT^over^ females compared to sham‐operated mice. *N* = 6. (C‐F) Plaque burden and insoluble amyloid beta (Aβ) in the cortex of 2‐month‐old *App^NL‐G‐F^* and *App^NL‐G‐F^*‐VAChT^over^ (C) sham‐operated and (D) OVX females, *N* = 3 to 10; and 3‐month‐old *App^NL‐G‐F^* and *App^NL‐G‐F^*‐VAChT^over^ (E) sham‐operated or (F) OVX females, *N* = 3 to 10. (G) Experimental timeline. (H) VAChT expression in the cortex of estradiol‐treated OVX‐*App^NL‐G‐F^* and OVX*‐App^NL‐G‐F^*‐VAChT^over^ 2‐month‐old females compared to placebo‐treated controls, *N* = 6. (I,J) Aβ immunofluorescence and insoluble Aβ in the cortex of (I) placebo‐treated or (J) estradiol‐treated 2‐month‐old *App^NL‐G‐F^* and *App^NL‐G‐F^*‐VAChT^over^ females, *N* = 6 to 8. VAChT‐KO samples were added as negative control. Synaptophysin (syn) was used as loading control. VAChT blot quantification is shown in Supplementary Figure 1. Scale bars = 1000 μm. Each symbol in the graphs represents an individual animal. One outlier value was excluded from the placebo‐treated dataset. Data expressed as mean ± SEM. Unpaired, two‐tailed Student *t* test. *p < 0.05.

Pathology in *App^NL‐G‐F^* mice develops early (∼2 months of age), and the hormonal status of the female mice reflects young women rather than postmenopausal women, who are the most affected by AD. To investigate whether the difference observed between male and female mice is due to female sex hormones, *App^NL‐G‐F^* and *App^NL‐G‐F^*‐VAChT^over^ females were depleted of sex hormones by OVX at the age of 1 month and assessed for amyloid pathology 1 and 2 months after surgery (Figure 3A). Efficacy of hormone manipulation was assessed by measuring dry uterine weights. OVX‐induced hormone depletion caused a significant decrease in uterine weight when compared to the sham condition (Figure S1A, B). VAChT levels were not affected by hormone manipulation, as sham‐operated and OVX females of the same genotype showed similar levels of VAChT expression, while 2‐ and 3‐month‐old *App^NL‐G‐F^*‐VAChT^over^ mice showed ∼twofold more VAChT than *App^NL‐G‐F^* controls (Figure 3B and Figure S1C, D).

Strikingly, at both 1 and 2 months after hormone depletion, plaque burden and insoluble Aβ levels were decreased in OVX‐*App^NL‐G‐F^*‐VAChT^over^ females when compared to OVX‐*App^NL‐G‐F^* controls (Figure 3D, F), suggesting that female sex hormones participate in the interplay between plaque pathology and VAChT levels. Notably, sham *App^NL‐G‐F^*‐VAChT^over^ and sham *App^NL‐G‐F^* females showed similar levels of amyloid buildup and insoluble Aβ levels (Figure 3C, E), reproducing our early findings (Figure 2).

As estradiol modulates cholinergic neurons in the brain,^17^, ^35^ we investigated whether estradiol treatment in OVX animals impacts the effect of increased VAChT expression on plaque pathology. One‐month‐old *App^NL‐G‐F^* and *App^NL‐G‐F^*‐VAChT^over^ females were ovariectomized and immediately treated with continuous estradiol or placebo for 1 month (Figure 3G). Estradiol‐treated females had higher uterine weight than placebo‐treated controls, indicating effectiveness of estradiol treatment (Figure S1E). Estradiol did not affect VAChT levels in the cortex (Figure 3H and Figure S1F). Conversely, estradiol prevented the protective effect of increased VAChT levels on plaque pathology observed in OVX mice (Figure 3I, J). These results indicate that estradiol influences how increased VAChT levels (and consequent increase in cholinergic signaling) regulate amyloid pathology.

### 3.3 VAChT deficiency exacerbates amyloid pathology in *App^NL‐F^* male but not in ovary‐intact *App^NL‐F^* female mice

**FIGURE 4:**
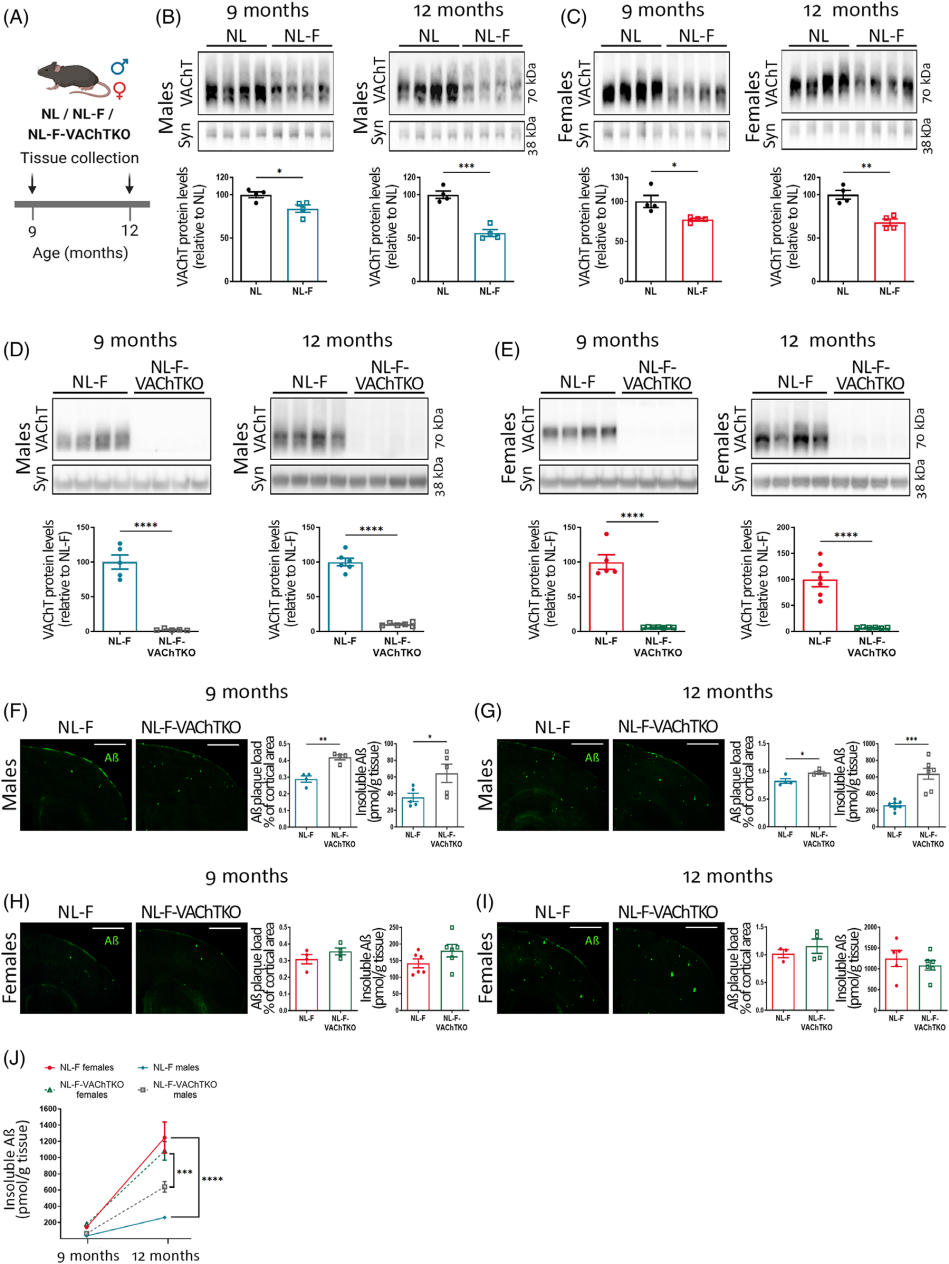
Vesicular acetylcholine transporter (VAChT) deficiency exacerbates amyloid pathology in *App^NL‐F^* males but not in ovary‐intact females. (A) Experimental timeline. (B,C) VAChT expression in the cortex of 9‐ and 12‐month‐old *App^NL‐F^* (B) males and (C) females compared to *App^NL^* controls. *N* = 4. (D,E) VAChT expression in the cortex of 9‐ and 12‐month‐old *App^NL‐F^‐*VAChT‐KO (D) males and (E) females compared to *App^NL‐F^* controls. *N* = 5 to 6. (F‐I) Plaque burden and insoluble amyloid beta (Aβ) in the cortex of 9‐ and 12‐month‐old *App^NL‐F^* and *App^NL‐F^*‐VAChT‐KO mice: (F) 9‐month‐old male and (G) 12‐month‐old male, *N* = 4 to 7; (H) 9‐month‐old female and (I) 12‐month‐old female, *N* = 3 to 6. (J) Insoluble Aβ over time (9 to 12 months) in *App^NL‐F^* and *App^NL‐F^*‐VAChT‐KO male and female mice, *N* = 5 to 7. Scale bars = 1000 μm. Synaptophysin (syn) was used as loading control. Each symbol in the graphs represents an individual animal. One outlier value was excluded from the 12‐month‐old female dataset. Data are expressed as mean ± SEM. Unpaired, two‐tailed Student *t* test and two‐way ANOVA adjusted with Sidak's multiple comparisons test for graph J. *p < 0.05, **p < 0.01, ***p < 0.001, ****p < 0.0001.

Amyloid pathology in *App^NL‐G‐F^* mice has an early onset and 6‐month‐old mice already exhibit extensive plaque buildup.^21^ To further study the sexual dimorphism in the cholinergic modulation of plaque pathology, we used the *App^NL‐F^* mouse model, in which pathology develops more slowly (starting at ∼9 months),^21^, ^36^ better mimicking the gradual progression of the human disease (Figure 4A). VAChT quantification in the cortex of 9‐ and 12‐month‐old *App^NL^* and *App^NL‐F^* mice showed that both male and female *App^NL‐F^* mice exhibited a decrease in cortical VAChT compared to *App^NL^* mice (Figure 4B, C). Thus, we tested whether further decrease in VAChT in the forebrain of *App^NL‐F^* mice exacerbates amyloid pathology by crossing *App^NL‐F^* mice with forebrain‐specific VAChT‐KO mice, which have the VAChT gene deleted selectively in forebrain cholinergic neurons.^6^, ^18^ In both males and females, VAChT is robustly decreased in *App^NL‐F^‐*VAChT‐KO mice (∼95% reduction) when compared to *App^NL‐F^* controls (Figure 4D, E). Nine‐ and 12‐month‐old *App^NL‐F^‐*VAChT‐KO males show a significant increase in amyloid burden and Aβ levels in the cortex compared to *App^NL‐F^* controls (Figure 4F, G), indicating that decreased VAChT expression in the forebrain of *App^NL‐F^* males exacerbates amyloid pathology. Conversely, the amount of both plaque deposition and insoluble amyloid were similar in VAChT‐deficient *App^NL‐F^* females compared to *App^NL‐F^* controls (Figure 4H, I). Strikingly, 9‐month‐old females of both genotypes showed three‐ to fourfold more insoluble Aβ than same‐age males. From 9 to 12 months, the accumulation of insoluble Aβ increased by around seven to nine times in both sexes and genotypes; however, accumulation was faster in females than in males (Figure 4J). Interestingly, while the accumulation rate of Aβ  in females of both genotypes was similar, in males, *App^NL‐F^‐*VAChT‐KO animals showed a faster buildup than *App^NL‐F^* counterparts. Thus, by 12 months, *App^NL‐F^* females had five times more Aβ than *App^NL‐F^* males, while *App^NL‐F^‐*VAChT‐KO females had two times higher levels than *App^NL‐F^‐*VAChT‐KO males. These results indicate that VAChT deficiency speeds up insoluble Aβ accumulation in males, while it has little or no impact on the rate of amyloid accumulation in ovary‐intact females.

### 3.4 Increased VAChT expression ameliorates amyloid pathology in *App^NL‐F^* males but not in ovary‐intact *App^NL‐F^* females

We then examined whether increasing VAChT levels in both male and female *App^NL‐F^* mice can mitigate Aβ pathology once Aβ aggregation has already started. To do that we used an AAV that carried the VAChT coding sequence (AAV‐VAChT) to increase VAChT expression in 9‐month‐old mice. We have shown that AAV‐VAChT leads to the expression of a functional protein that increases the secretion of ACh.^37^ We tested the ability of the AAV‐VAChT vector to induce VAChT overexpression in the cortex of forebrain‐specific VAChT‐KO mice, which served as a null background. Forebrain‐specific VAChT‐KO mice injected with AAV‐VAChT in the vertical limb of the diagonal band exhibit substantial VAChT staining in the basal forebrain and cortical neuronal projections (Figure S2B), as opposed to forebrain‐specific VAChT‐KO mice that received AAV‐mCherry control (Figure S2A).

**FIGURE 5:**
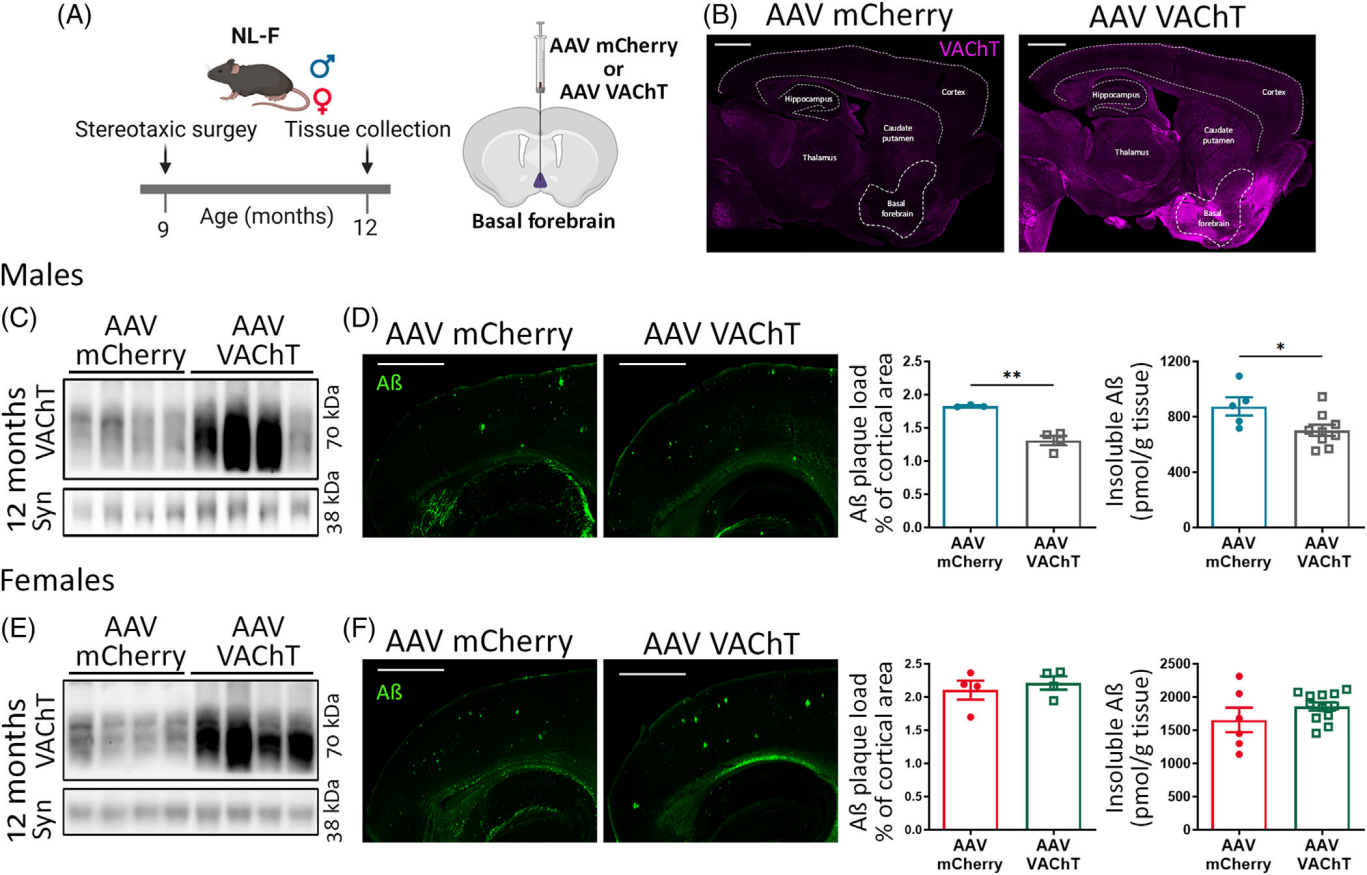
Increased vesicular acetylcholine transporter (VAChT) expression ameliorates amyloid pathology in *App^NL‐F^* males but not in ovary‐intact females. (A) Experimental timeline. (B) VAChT immunostaining in sagittal sections. (C) VAChT expression in the cortex of adeno‐associated virus (AAV)‐VAChT‐injected *App^NL‐F^* males compared to AAV‐mCherry‐injected controls, *N* = 5 to 8. (D) Analysis of plaque burden and insoluble amyloid beta (Aβ) in the cortex of *App^NL‐F^* males injected with AAV‐mCherry or AAV‐VAChT, *N* = 3 to 8. (E) VAChT expression in the cortex of AAV‐VAChT‐injected *App^NL‐F^* females compared to AAV‐mCherry‐injected controls, *N* = 6 to 12. (F) Plaque burden and insoluble Aβ in the cortex of *App^NL‐F^* females injected with AAV‐mCherry or AAV‐VAChT, *N* = 4 to 12. Synaptophysin (syn) was used as loading control. Quantification of VAChT blots is shown in Supplementary Figure 2. Scale bars = 1000 μm. Each symbol in the graphs represents an individual animal. One outlier value was excluded from the male dataset. Data are expressed as mean ± SEM. Unpaired, two‐tailed Student *t* test. *p < 0.05, **p < 0.01.

Next, we injected 9‐month‐old male and female *App^NL‐F^* mice with AAV‐VAChT (or AAV‐mCherry) in the vertical limb of the diagonal band and assessed Aβ accumulation 3 months after injection (Figure 5A). Immunofluorescence showed strong VAChT labeling at the site of injection in *App^NL‐F^* mice that received AAV‐VAChT, as opposed to those that received AAV‐mCherry (Figure 5B). Moreover, immunoblot quantification demonstrated that VAChT levels were increased ∼2‐fold in the cortex of *App^NL‐F^* males and females injected with AAV‐VAChT when compared to AAV‐mCherry controls (Figure 5C, E and Figure S2C, D). Importantly, while overexpression of VAChT led to decreased plaque area and insoluble Aβ levels in the cortex of *App^NL‐F^* males (Figure 5D), Aβ pathology was not altered in ovary‐intact *App^NL‐F^* females with increased VAChT levels (Figure 5F). These findings suggest that VAChT levels have a causal and inverse relationship with amyloid pathology and that increased VAChT levels can mitigate pathology even when aggregation has already occurred. Also, these results further support the causal relationship between VAChT levels and amyloid pathology in male mice and demonstrate that ovary‐intact females do not exhibit the regulatory function of cholinergic signaling in amyloid pathology.

### 3.5 Cholinergic dysfunction worsens amyloid pathology in elderly men and postmenopausal women

To investigate whether cholinergic dysfunction has a sex‐dependent association with amyloid pathology in humans, we used structural MRI and amyloid PET scans from the AIBL Study of Ageing^24^ to perform a comparative longitudinal analysis on males and females. Because basal forebrain degeneration occurs in presymptomatic stages of AD,^29^, ^38^ we selected cognitively normal older adults with at least two timepoints of 3Tesla T1 weighted structural MRI data, and amyloid PET imaging using the same tracer type (PIB, AV45, NAV, or Flutemetamol). In total, 130 individuals were included (58 males, 72 females; mean age: 75 years). Male and female participants did not differ on median age, percentage of converts to mild cognitive impairment (MCI) or AD, percentage of *APOE*4 carriers, or interval in years between MRI and PET scans (Table 1). Whole brain main effects of sex (male, female) on longitudinal amyloid PET APC were investigated using a flexible factorial ANCOVA model (see Methods).

**FIGURE 6:**
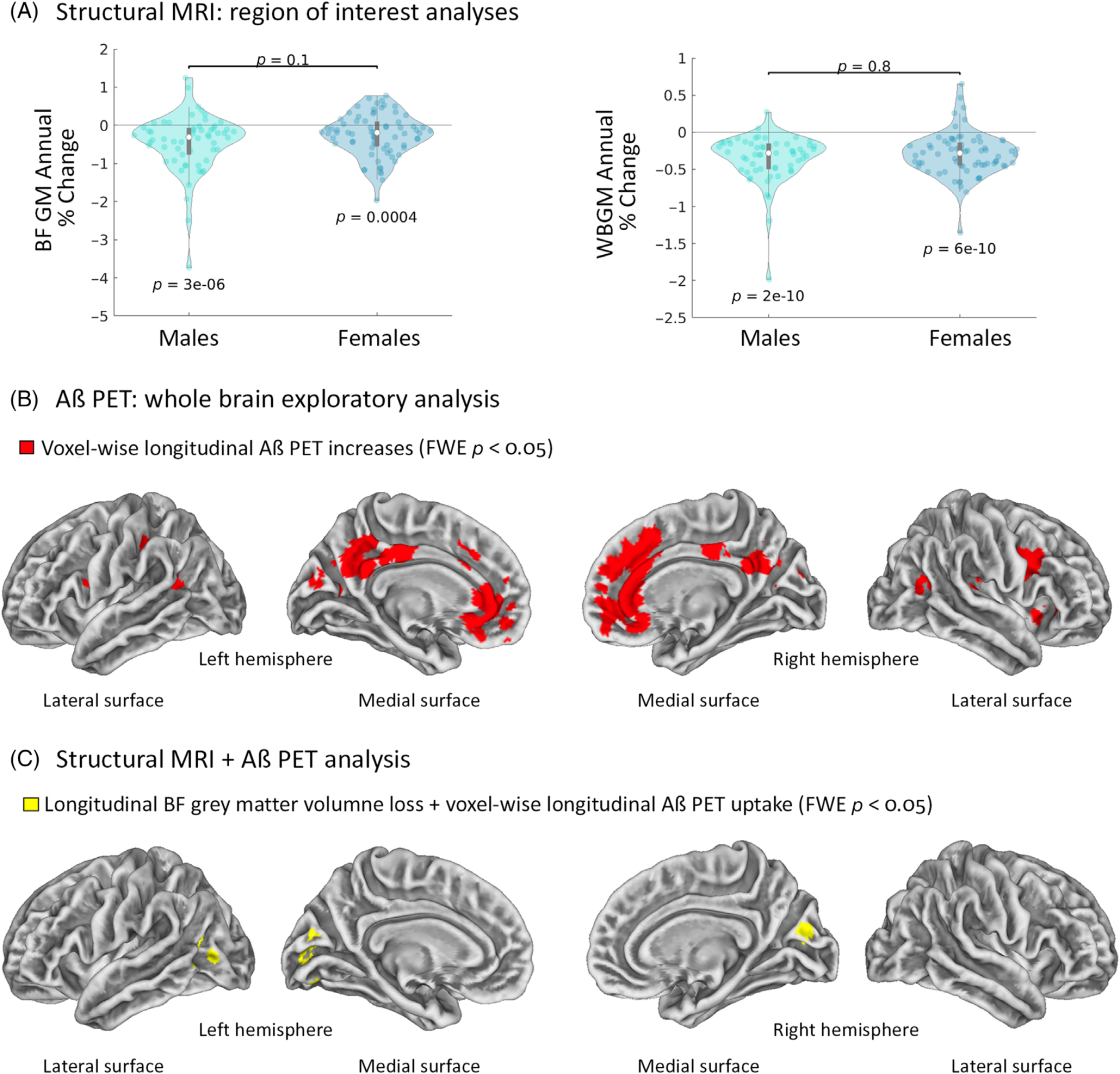
Cholinergic dysfunction worsens amyloid pathology in humans independently of biological sex. (A) Degeneration of grey matter (GM) regions of interest, as measured by longitudinal structural magnetic resonance imaging (MRI) data. Annual percent change (APC) for the posterior basal forebrain (BF GM; left) and whole brain grey matter (WB GM; right) in males and females. Negative APC values indicate longitudinal grey matter loss. *p* values below violin plots show whether median APC differs from zero (*N* = 58 males, 72 females). (B,C) Three‐dimensional cortical surfaces of each brain hemisphere viewed from both the medial and lateral surfaces. Statistics were conducted in volumetric space but projected onto a cortical surface for visualization. No sex differences were detected, thus participants of both sexes were pooled in the analysis (*N* = 130). (B) Voxel‐wise longitudinal Aβ positron emission tomography (PET) analysis. Red‐labeled voxels show significant increase in Aβ after familywise error rate (FWER) correction for multiple comparisons across the whole brain grey matter. (C) Voxel‐wise longitudinal correlations between structural MRI (APC of the basal forebrain region of interest) and Aβ PET for each participant. Yellow‐labelled voxels show where greater grey matter loss in the basal forebrain correlates with greater increase in Aβ tracer uptake in PET.

Analysis of longitudinal rates of grey matter loss, measured by APC, showed that both males and females exhibited significant longitudinal grey matter degeneration of the posterior basal forebrain (Ch4 and Ch4p) (Figure 6A, left panel). No sex difference was observed in longitudinal posterior basal forebrain APC (*p* = 0.1). Moreover, we found that males and females do not differ in whole brain grey matter APC (*p* = 0.8; Figure 6A; right panel).

Next, we assessed longitudinal patterns of whole brain amyloid uptake, as measured by PET. We observed a significant increase in brain amyloid over time throughout the cortical midline network in the pooled cohort (Figure 6B), but no main effect of sex on longitudinal amyloid PET was detected, even when using a more liberal threshold of *p* < 0.001 (uncorrected). Thus, although longitudinal increases in brain amyloid were evident in the cohort, the magnitude and distribution of these changes did not differ by biological sex.

As previous works indicate that basal forebrain degeneration relates to pathology in cortical targets,^28^, ^39^ we investigated the relationship between longitudinal posterior basal forebrain degeneration and longitudinal amyloid accumulation. We used an ANCOVA model with the sMRI estimates of longitudinal basal forebrain degeneration APC as the predictor, and voxel‐wise PET estimates of longitudinal amyloid accumulation APC as the dependent measure. The posterior basal forebrain predictor was split by sex, enabling us to examine if the relationship (slope or magnitude) of posterior basal forebrain degeneration and amyloid accumulation differed between males and females. We found that longitudinal basal forebrain degeneration correlated with increased amyloid deposition in bilateral occipital‐temporal regions (FWER *p* < 0.05, Figure 6C) but no significant moderating effects of sex were detected, even at more liberal thresholds (uncorrected *p* < 0.001). These data indicate that in elderly humans, biological sex does not significantly alter longitudinal trajectories of grey matter atrophy and amyloid pathology, or their relationship.

## 4 DISCUSSION

Data from preclinical and clinical studies support sex‐specific differences in AD pathophysiology. However, basic research still highly focuses on male animal models, hindering understanding of the role of sex in AD development and progression.^40^ This sex bias also contributes to poor translation and replicability in preclinical studies.^41^

In this translational study, we revealed that biological sex impacts the inverse relationship between cholinergic signaling and amyloid pathology in mouse models. Moreover, we observed that estradiol disrupts the modulation of Aβ pathology by the cholinergic system. In elderly humans, the relationship between amyloid pathology and cholinergic tone in postmenopausal women closely resembled that of their male counterparts, suggesting that the effects of estradiol are no longer evident in older individuals.

Aβ negatively impacts cholinergic signaling by reducing ACh synthesis and release,^16^ impairing nicotinic and muscarinic receptor signaling,^42^, ^43^ inducing cholinergic neurons toxicity^44^ and decreasing VAChT expression in vitro and in vivo.^45^, ^46^, ^47^ Our data show decreased VAChT levels in *App^NL‐G‐F^* and *App^NL‐F^* mice, supporting and expanding these reports and indicates that VAChT change is an early pathological event. These findings align with studies in AD patients, where VAChT levels inversely correlated with pathology and cognitive deficits.^6^, ^8^ Notably, forebrain‐specific‐VAChT‐KO mice display increased hippocampal Aβ^6^ but no plaques, due to weak murine amyloid aggregation.^48^

We found that increasing VAChT levels in *App^NL‐G‐F^* or *App^NL‐F^* male mice, either by transgene or by viral expression, reduces Aβ pathology. VAChT overexpression facilitates ACh release in the brain^20^ by increasing ACh accumulation in synaptic vesicles.^32^ This cholinergic boost may regulate Aβ levels through various mechanisms. ACh signaling influences microRNA and RNA metabolism with global consequences for gene expression and alternative splicing.^6^, ^49^ Key AD‐associated transcripts are affected^6^ through M1 muscarinic ACh receptor (mAChR) activation,^19^ including the mRNA for β‐secretase. Notably, M1 activation promotes non‐amyloidogenic APP processing, reducing Aβ synthesis.^50^, ^51^ Nicotinic ACh receptor stimulation is also protective, as it enhances microglial Aβ phagocytosis, reducing amyloid burden.^52^, ^53^ ACh also increases and stabilizes the soluble Aβ conformation.^54^ Moreover, cholinergic signaling is anti‐inflammatory and antioxidant.^53^, ^55^ Evidence suggests a potential association between cholinergic basal forebrain and inflammatory response to Aβ in AD.^56^

In contrast to males, increased VAChT levels did not decrease amyloid pathology in ovary‐intact females in both *App^NL‐G‐F^* and *App^NL‐F^* mice, indicating a sex‐specific cholinergic influence on Aβ pathology. Sex differences involving the cholinergic system have been studied in various models. Rodents show sexual dimorphism in basal forebrain cholinergic neuron size and numbers, region‐specific ACh release, and age‐related cholinergic decline.^17^ Additionally, sex‐dependent changes in transfer‐RNA‐fragments (tRFs), which are non‐coding RNA regulators, have been reported in AD. tRFs that modulate cholinergic transcripts in the nucleus accumbens may potentially lead to accelerated cognitive decline in females with AD.^57^ Furthermore, sex hormones influence numerous neural/behavioral functions. Basal forebrain cholinergic neurons express estrogen receptors,^58^ and estradiol regulates cholinergic neurotransmission by increasing choline acetyltransferase levels in the basal forebrain and hippocampal ACh release.^17^

Interestingly, VAChT‐overexpression reduced Aβ pathology in OVX‐*App^NL‐G‐F^* females, an effect prevented by estradiol, demonstrating that estradiol uncouples cholinergic signaling regulation of Aβ pathology. While estrogens mitigate some aspects of inflammation,^59^ and affect brain mAChR expression,^60^ the specific mechanism by which estradiol regulates cholinergic influence on amyloid accumulation remains undetermined.

The relationship between cholinergic deficiency, Aβ levels, and AD severity is well documented.^6^, ^8^, ^51^ Correspondingly, near‐complete VAChT deletion in *App^NL‐F^* male mice forebrain worsened plaque pathology, an effect not observed in ovary‐intact females. These results, along with overexpression experiments, provide strong evidence for a causal, bidirectional relationship between VAChT levels and amyloid pathology in male mice and suggest that, in young female mice, estradiol dissociates Aβ pathology from cholinergic signaling.

Notably, both *App^NL‐G‐F^* and *App^NL‐F^* sham‐operated mice that underwent surgical procedures presented higher insoluble Aβ levels than intact mice of the same age/sex/genotype (compare Figure 3C/E with Figure 2F/G, respectively, and Figure 5D/F with Figure 4G/I). Although the mechanism is unclear, these results agree with preclinical evidence suggesting that volatile anesthetics used in surgeries may be a risk factor for AD as they aggravate Aβ pathology.^61^, ^62^

Women are at higher risk of developing AD and often progress to more severe symptoms than men.^2^, ^3^ Strikingly, we observed no sex differences in longitudinal amyloid accumulation in ∼75‐year‐olds. Similarly, a study of cognitively normal individuals (70‐ to 96‐year‐olds) did not detect sex differences in amyloid PET.^63^ However, sex differences in amyloid pathology in elderly individuals are controversial. Worse amyloid PET pathology was reported in men in cohorts 70 to 85 years old,^64^ while in cohorts of ∼64‐year‐olds^65^ and 40‐ to 60‐year‐olds,^66^ women exhibited worse amyloid pathology, particularly during menopause transition. It is tempting to speculate that sexual dimorphism in amyloid pathology may vary depending on age. Whether menopause drives this nonlinear variation requires further study.

No sex differences in grey matter decrease rates was observed in individuals over 70 years.^67^, ^68^ We found similar results in posterior basal forebrain degeneration and whole brain grey matter annual change. Parallel structural MRI analyses suggest that grey matter integrity between men and women is more differentiable in early midlife than in later life, potentially mirroring the age‐dependent effects observed with amyloid PET.^67^, ^68^

Importantly, our findings are consistent with prior studies,^14^, ^69^, ^70^ where decreasing basal forebrain grey matter volume correlates with increasing amyloid burden. However, no sex differences were observed for this relationship in this advanced age range. Notably, all women in our cohort were ∼2 decades postmenopausal, time in which the dissociation of cholinergic signaling and amyloid pathology by estradiol should be absent. Our observations highlight the need for large multimodal longitudinal sMRI and Aβ‐PET studies covering ages 40 to 60.

In many mouse models of AD, pathology develops at young age and females usually do not depict the postmenopausal hormonal changes observed in women.^71^ Our results suggest that ovariectomized females better mimic aged women, as ovary removal lifts the estradiol‐dependent disruption of the association between amyloid pathology and cholinergic signaling. Possibly, to fully model pathological changes in women, female AD mouse models need to replicate postmenopausal hormonal status. This may be a crucial improvement for understanding sex as a biological variable in AD models.

Moreover, our data highlight the potential impact of hormone replacement therapy (HRT) on AD management in postmenopausal women. We show that estrogen decouples the relationship between VAChT levels and amyloid pathology, raising the possibility that HRT could interfere with the potential benefits of cholinesterase inhibitors (ChEIs; drugs inhibiting ACh breakdown) prescribed to AD patients. Preclinical studies suggest that sex differences might modify ChEIs’ pharmacological effects.^17^ However, the impact of sex on ChEIs’ efficacy and tolerability is often overlooked.^17^ Most studies do not report data by sex and the available results are conflicting.^72^ Some describe a more selective benefit of ChEIs in men,^73^ others in women^74^ or no significant sex differences.^72^ Likewise, HRT's influence in AD is equally controversial. While certain studies suggest postmenopausal estrogen is protective against AD, others dispute this beneficial effect,^75^ even suggesting a higher dementia risk in postmenopausal women given estrogen.^75^ Complicating factors may explain these contradictory results, including HRT type, duration, and initiation timing, and *APOE* genotype.^75^ Understanding sex differences and HRT's influence on ChEIs could improve treatment benefits, help dosage adjustments, and identify responders versus non‐responders.^17^ Together, these results highlight the need of considering age and sex when prescribing cholinergic‐targeting drugs.

This study has some limitations. OVX was performed in 1‐month‐old mice, just before the onset of the estrous cycle, to avoid confounding effects of endogenous hormonal levels. However, menopause occurs after years of menstrual cycles and hormonal fluctuations. Hence, an aging model that reproduces hormonal changes in women after menopause may be warranted. Additionally, the study in young females overlooks the possible impact of aging, and we did not evaluate cognitive function in our analyses, which could have linked pathology to dementia symptoms. Furthermore, we used basal forebrain volume as a proxy for cholinergic signaling in humans, and future analyses should correlate VAChT levels determined using PET with amyloid pathology.

In summary, our data reveal a causal and bidirectional relationship between VAChT levels and amyloid pathology in male and ovariectomized female mice, which more closely represent postmenopausal women. The lack of cholinergic regulation of Aβ deposition in ovary‐intact female mice is due to estradiol dissociating amyloid pathology from cholinergic signaling. In humans ∼75 years old there are no sex differences in Aβ deposition, forebrain atrophy, or the relationship between these measures. These results emphasize the need to consider the hormonal context and its potential interactions with the cholinergic system and amyloid pathology in mice, as well as the translatability of results between ovary‐intact female mice and postmenopausal women. The implications of our study are broad, given that rarely human hormonal status is reproduced in mice. We propose that estradiol suppression is necessary to replicate the hormonal situation of postmenopausal women to faithfully study AD and likely other age‐related neurodegenerative diseases.

## CONFLICT OF INTEREST STATEMENT

The authors have no conflicts to disclose. Author disclosures are available in the supporting information.

## CONSENT STATEMENT

All participants provided written informed consent before participation.

Figure 1. https://figshare.com/s/a12a24d5da59d20bcabb

Figure 2. https://figshare.com/s/a2374fd2d328a3ee4c8e

Figure 3. https://figshare.com/s/5bd72279fbfba0a4ab67

Figure 4. https://figshare.com/s/8913adb0987c0797acbe

Figure 5. https://figshare.com/s/ffc6b5ae310771bf896a

## Supporting information

Supporting Information

Supporting Information

## Data Availability

Data supporting findings of this study are available in figshare at the following links:

## 1

Collaborators associated with the AIBL research group

Arti Appannah, Mary Barnes, Kevin Barnham, Justin Bedo, Shayne Bellingham, Lynette Bon, Pierrick Bourgeat, Belinda Brown, Rachel Buckley, Samantha Burnham, Ashley Bush, Graeme Chandler, Karren Chen, Roger Clarnette, Steven Collins, Ian Cooke, Tiffany Cowie, Kay Cox, Emily Cuningham, Elizabeth Cyarto, Phuong Anh Vu Dang, David Darby, Patricia Desmond, James Doecke, Vincent Dore, Harriet Downing, Belinda Dridan, Konsta Duesing, Michael Fahey, Maree Farrow, Noel Faux, Michael Fenech, Shane Fernandez, Binosha Fernando, Chris Fowler, Maxime Francois, Jurgen Fripp, Shaun Frost, Samantha Gardener, Simon Gibson, Petra Graham, Veer Gupta, David Hansen, Karra Harrington, Andy Hill, Eugene Hone, Maryam Hor, Malcolm Horne, Brenda Huckstepp, Andrew Jones, Gareth Jones, Adrian Kamer, Yogi Kanagasingam, Lisa Keam, Adam Kowalczyk, Betty Krivdic, Chiou Peng Lam, Fiona Lamb, Nicola Lautenschlager, Simon Laws, Wayne Leifert, Nat Lenzo, Hugo Leroux, Falak Lftikhar, Qiao‐Xin Li, Florence Lim, Lucy Lim, Linda Lockett, Kathy Lucas, Mark Mano, Caroline Marczak, Georgia Martins, Paul Maruff, Yumiko Matsumoto, Sabine Bird, Rachel McKay, Rachel Mulligan, Tabitha Nash, Julie Nigro, Graeme O'Keefe, Kevin Ong, Bernadette Parker, Steve Pedrini, Jeremiah Peiffer, Sveltana Pejoska, Lisa Penny, Keyla Perez, Kelly Pertile, Pramit Phal, Tenielle Porter, Stephanie Rainey‐Smith, Parnesh Raniga, Alan Rembach, Carolina Restrepo, Malcolm Riley, Blaine Roberts, Jo Robertson, Mark Rodrigues, Alicia Rooney, Rebecca Rumble, Tim Ryan, Mather Samuel, Ian Saunders, Greg Savage, Brendan Silbert, Hamid Sohrabi, Julie Syrette, Cassandra Szoeke, Kevin Taddei, Tania Taddei, Sherilyn Tan, Michelle Tegg, Philip Thomas, Darshan Trivedi, Brett Trounson, Robyn Veljanovski, Giuseppe Verdile, Victor Villemagne, Irene Volitakis, Cassandra Vockler, Michael Vovos, Freda Vrantsidis, Stacey Walker, Andrew Watt, Mike Weinborn, Bill Wilson, Michael Woodward, Olga Yastrubetskaya, Paul Yates, Ping Zhang
