# Supplementary material for: Sex‐dependent cholinergic effects on amyloid pathology: A translational study
Source: Alzheimers Dement. 2023 Oct 17;20(2):995–1012. doi: 10.1002/alz.13481 (PMC10916951; doi:10.1002/alz.13481)
Supplement: Supplementary file 1 — Supporting Information [file ALZ-20-995-s002.pdf]

Supplementary table 1. Insoluble Aβ (pmol/g tissue) in the cortex of *App*$^{NL\text{-}G\text{-}F}$ and *App*$^{NL\text{-}G\text{-}F}$-VAChT^{over} males and females.

	***App*$^{NL\text{-}G\text{-}F}$**			***App*$^{NL\text{-}G\text{-}F}$-VAChT^{over}**		
	Aβ males Mean (SEM)	Aβ females Mean (SEM)	p-value	Aβ males Mean (SEM)	Aβ females Mean (SEM)	p-value
2 months	173.3 (19.93)	150.9 (12.24)	0.3598	122.9 (5.476)	142.5 (10.71)	0.1331
3 months	477.7 (30.59)	389.2 (32.27)	0.0814	373.4 (19.37)	343.6 (30.92)	0.458
6 months	1019 (63.37)	827.3 (72.66)	0.0843	1070 (67.92)	838.3 (79.21)	0.0581

p-value was determined by unpaired, two-tailed t-test

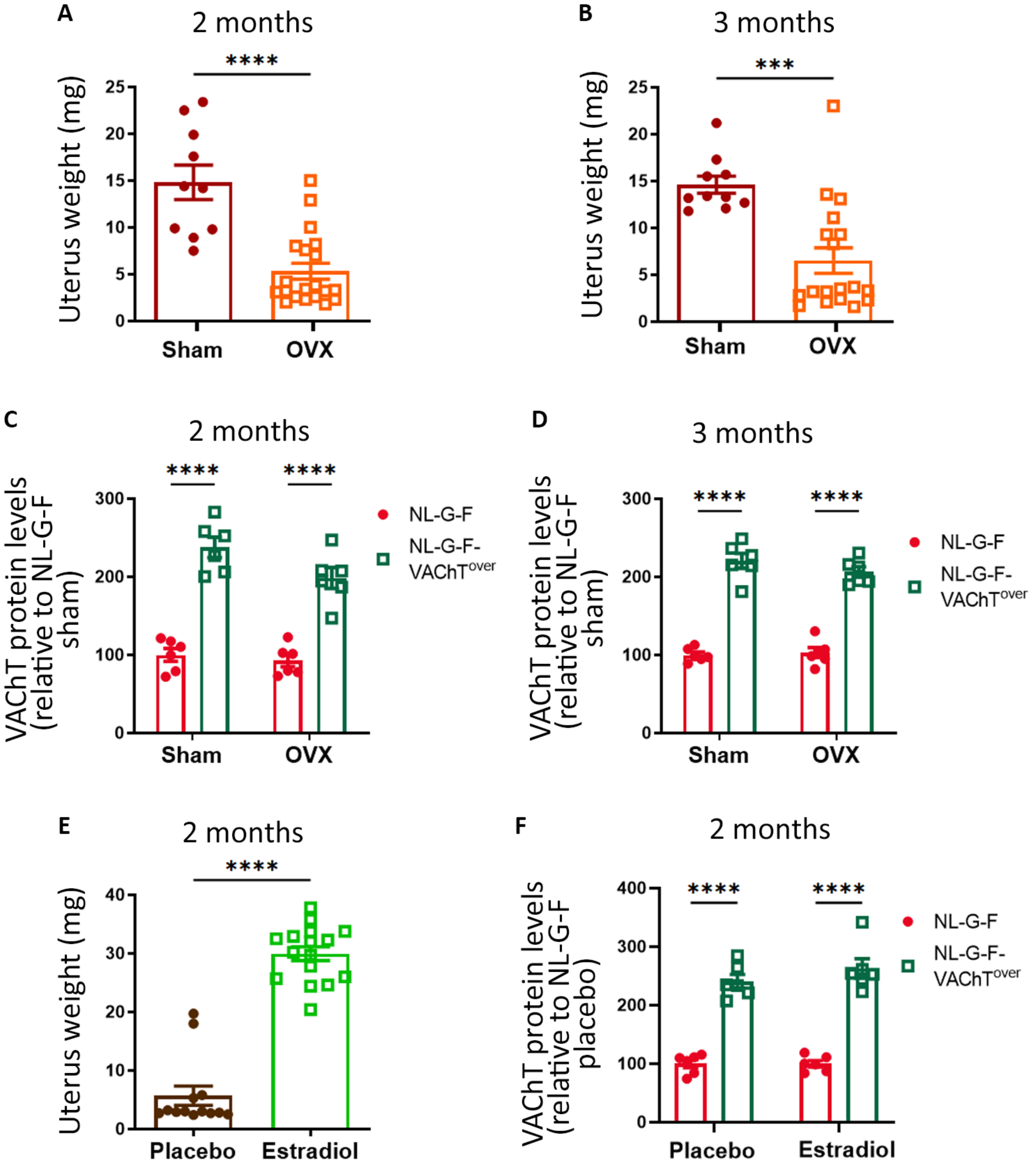

Fig. S1. Uterus weight and cortical VAChT levels in *App*$^{NL\text{-}G\text{-}F}$ and *App*$^{NL\text{-}G\text{-}F}$-VAChT^{over} mice. (**A** and **B**) Uterus weight in sham-operated and ovariectomized females in (A) 2- and (B) 3-month-old mice. No significant difference in uterine weights between genotypes (sham *App*$^{NL\text{-}G\text{-}F}$ vs sham *App*$^{NL\text{-}G\text{-}F}$-VAChT^{over} at 2 months p=0.5228 and 3 months p=0.9944; OVX *App*$^{NL\text{-}G\text{-}F}$ vs OVX *App*$^{NL\text{-}G\text{-}F}$-VAChT^{over} at 2 months p=0.1272 and 3 months p=0.9960) so the weight data was

grouped by surgical procedure. *N*=10 sham, *N*=20 OVX. (**C** and **D**) VAChT protein levels (normalized to synaptophysin) in *App*$^{NL\text{-}G\text{-}F}$ and *App*$^{NL\text{-}G\text{-}F}$-VAChT^{over} ovariectomized and sham-operated females at (C) 2 and (D) 3 months of age. *N*=6. (**E**) Uterus weight in 2-month-old ovariectomized *App*$^{NL\text{-}G\text{-}F}$ and *App*$^{NL\text{-}G\text{-}F}$-VAChT^{over} females that received placebo or estradiol. No significant difference in uterine weight between genotypes (placebo *App*$^{NL\text{-}G\text{-}F}$ vs placebo *App*$^{NL\text{-}G\text{-}F}$-VAChT^{over} *p*=0.4275; estradiol *App*$^{NL\text{-}G\text{-}F}$ vs estradiol *App*$^{NL\text{-}G\text{-}F}$-VAChT^{over} *p*=0.9367), so data was pooled together. *N*=13 placebo, *N*=16 estradiol. (**F**) VAChT protein levels (normalized to synaptophysin) in 2-month-old *App*$^{NL\text{-}G\text{-}F}$ and *App*$^{NL\text{-}G\text{-}F}$-VAChT^{over} ovariectomized females that received placebo or estradiol. *N*=6. Data expressed as mean ± SEM. Unpaired, two-tailed student's t test for graphs A, B, E, and two-way ANOVA adjusted with Sidak's multiple comparisons test for graphs C, D, F. ***p<0.001, ****p<0.0001.

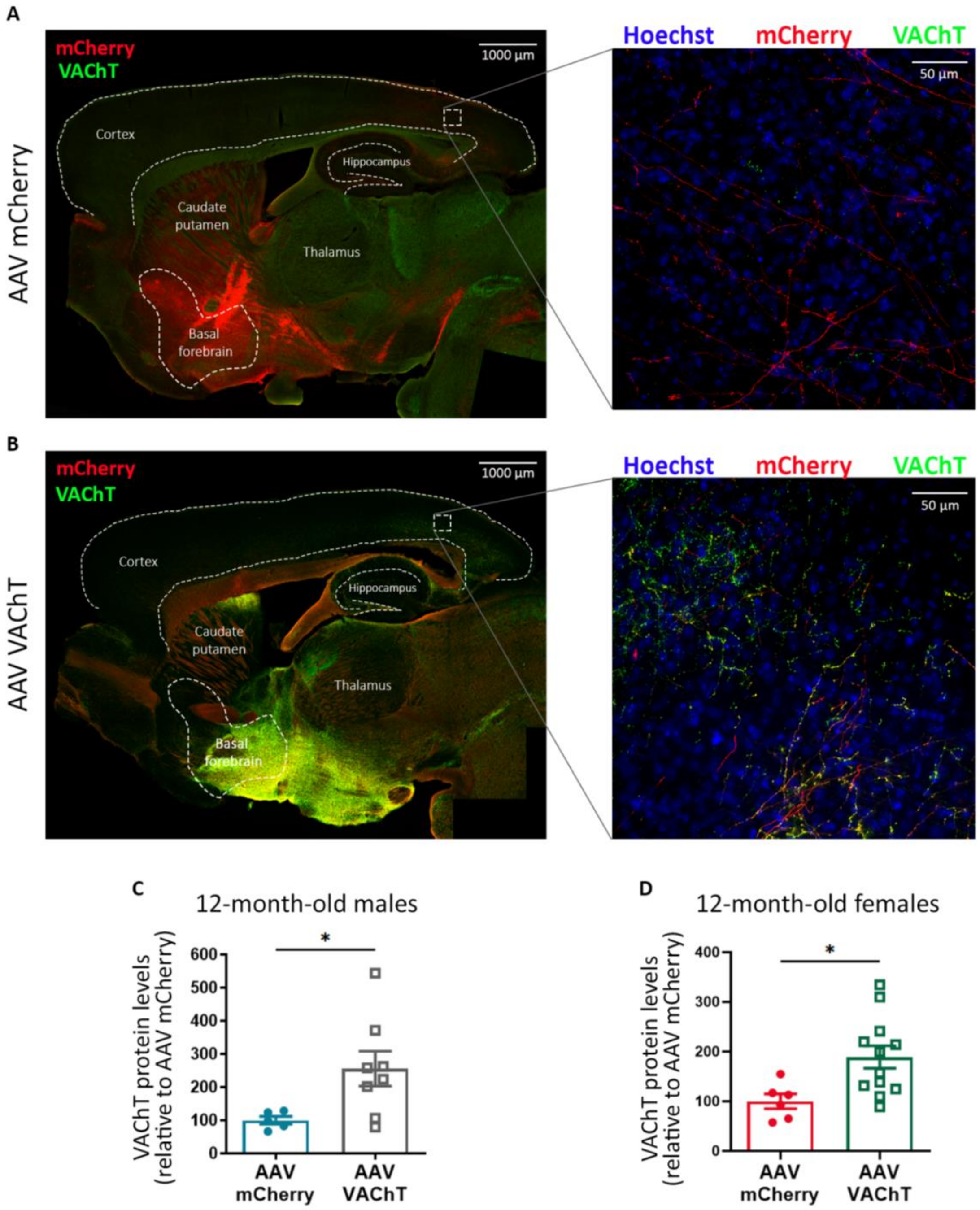

Fig. S2. Overexpression of VAChT by AAV injection. (**A** and **B**) Immunofluorescence images of VAChT-KO mice that received (A) AAV-mCherry or (B) AAV-VAChT into the basal forebrain. Nuclei are shown in blue, mCherry in red and VAChT in green. A close-up of the cortex is shown. (**C** and **D**) VAChT protein levels (normalized to synaptophysin) in 12-month-old (C)

male and (D) female $App^{NL\text{-}F}$ mice that received either AAV-mCherry or AAV-VAChT injections into the basal forebrain. N=5-7 for males and N=6-12 for females. Data are expressed as mean ± SEM and analyzed using unpaired, two-tailed student's t test. *$p<0.05$.
